# Supplementary material for: Discovery of genes required for body axis and limb formation by global identification of retinoic acid–regulated epigenetic marks
Source: PLoS Biol. 2020 May 18;18(5):e3000719. doi: 10.1371/journal.pbio.3000719 (PMC7259794; doi:10.1371/journal.pbio.3000719)
Supplement: S1 Table — Aldh1a2, aldehyde dehydrogenase 1A2; ChIP-seq, chromatin immunoprecipitation sequencing; E, embryonic day; H3K27ac, histone H3 K27 acetylation; RA, retinoic acid. (DOCX) [file pbio.3000719.s001.docx]

S1 Table. Comparison of *Aldh1a2-/-* and wild-type E8.5 trunk tissue for H3K27ac ChIP-seq and RNA-seq results to identify RA-regulated H3K27ac ChIP-seq peaks near genes with RA-regulated expression.

| **H3K27ac ChIP-seq differential peak for *Aldh1a2* KO vs WT**  **(mm10)** | **log2 fold change: H3K27ac ChIP-seq for *Aldh1a2* KO vs WT** | **RARE: based on Homer TFBS analysis** | **nearby gene with altered expression in *Aldh1a2* KO vs WT** | **log2 fold change for nearby gene:**  **RNA-seq for**  ***Aldh1a2* KO vs WT** |
| --- | --- | --- | --- | --- |
| chr13:78197222-78204291 | -1.23 | DR1 | Nr2f1 | -2.02 |
| chr13:34133342-34134366 | -1.13 | - | Tubb2b | -1.03 |
| chr18:83838984-83841358 | -1.10 | DR5, DR2 | Tshz1 | -1.33 |
| chr11:68049983-68051745 | -1.07 | DR2, DR1 | Stx8 | -2.87 |
| chr6:84975748-84978033 | -1.03 | DR5 | Zfp638 | -1.33 |
| chr8:14991303-14993068 | -0.96 | - | Arhgef10 | -1.56 |
| chr11:68088685-68091257 | -0.90 | - | Stx8 | -2.87 |
| chr17:56468893-56471601 | -0.90 | DR2, DR1 | Ptprs * | -3.31 |
| chr9:63715651-63717278 | -0.89 | DR5 | Smad3 | -2.09 |
| chr19:21164673-21168435 | -0.86 | DR2 | Zfand5 | -2.37 |
| chr9:16152232-16154557 | -0.86 | - | Fat3 | -3.02 |
| chr9:118653618-118656175 | -0.84 | DR5, DR1 | Itga9 | -1.63 |
| chr6:5028587-5031108 | -0.83 | DR5, DR2 | Ppp1r9a | -1.47 |
| chr9:114798509-114801621 | -0.81 | - | Cmtm8 | -2.12 |
| chr4:149254383-149256741 | -0.80 | - | Kif1b | -1.72 |
| chr5:36373121-36374553 | -0.79 | DR5 | Sorcs2 | -5.20 |
| chr3:5387155-5389128 | -0.79 | DR5 | Zfhx4 * | -2.26 |
| chr17:30467016-30471968 | -0.78 | DR1 | Btbd9 | -1.18 |
| chr3:5235978-5239655 | -0.77 | - | Zfhx4 * | -2.26 |
| chr9:35442832-35445303 | -0.74 | - | Cdon | -1.60 |
| chr14:98034746-98040239 | -0.71 | DR5, DR1 | Dach1 | -2.98 |
| chr6:14897860-14903160 | -0.67 | DR1 | Foxp2 | -1.26 |
| chr16:44529549-44532004 | -0.67 | - | Boc | -0.87 |
| chr14:52330133-52333035 | -0.67 | - | Sall2 | -1.17 |
| chr4:145033496-145035860 | -0.65 | DR5 | Dhrs3 * | -1.11 |
| chr12:8912431-8914944 | -0.64 | - | Laptm4a | -1.97 |
| chr18:83927674-83929824 | -0.64 | - | Tshz1 | -1.33 |
| chr14:14069903-14072065 | -0.64 | - | Atxn7 | -1.50 |
| chr5:111242680-111244777 | -0.63 | DR1 | Ttc28 | -1.87 |
| chr17:66410064-66414806 | -0.63 | DR5, DR1 | Mtcl1 | -1.32 |
| chr4:148106909-148109328 | -0.63 | - | Draxin | -3.43 |
| chr14:16571405-16576397 | -0.63 | DR5 | Rarb * | -1.64 |
| chr16:74395975-74399535 | -0.63 | DR1 | Robo2 | -1.22 |
| chr18:84069541-84075594 | -0.62 | DR5 | Tshz1 | -1.33 |
| chr11:18962656-18965461 | -0.61 | DR5, DR1 | Meis1 * | -2.64 |
| chr9:96989027-96991630 | -0.60 | DR5 | Spsb4 | -2.23 |
| chr3:108409804-108412280 | -0.60 | - | Celsr2 | -7.29 |
| chr17:47897351-47899993 | -0.59 | DR1 | Foxp4 * | -1.02 |
| chr14:78848672-78851835 | -0.58 | DR5, DR2, DR1 | Vwa8 | -1.08 |
| chr2:105689278-105690982 | -0.58 | - | Pax6 | -3.02 |
| chr3:87956774-87961235 | -0.58 | DR2, DR1 | Crabp2 | -2.82 |
| chr2:116019003-116024272 | -0.58 | DR2 | Meis2 * | -1.10 |
| chr4:107834342-107836683 | -0.58 | DR2 | Lrp8 | -1.54 |
| chr7:70348715-70369942 | -0.57 | DR1 | Nr2f2 * | -2.32 |
| chr11:18956989-18958835 | -0.57 | DR5 | Meis1 * | -2.64 |
| chr13:34129519-34132640 | -0.55 | DR2 | Tubb2b | -1.03 |
| chr11:19012000-19025444 | -0.54 | DR1 | Meis1 * | -2.64 |
| chr9:96956410-96959728 | -0.54 | DR2 | Spsb4 | -2.23 |
| chr9:48692264-48699040 | -0.54 | DR2, DR1 | Zbtb16 | 1.36 |
| chr3:34678267-34680699 | -0.54 | DR2 | Sox2 | -0.86 |
| chr6:144250107-144252835 | -0.52 | - | Sox5 | -2.33 |
| chr18:61033064-61036494 | -0.52 | DR2, DR1 | Cdx1 | -2.00 |
| chr3:34647848-34655776 | -0.51 | - | Sox2 | -0.86 |
| chr17:56475307-56476820 | -0.51 | - | Ptprs * | -3.30 |
| chr7:133035031-133040386 | -0.51 | DR2 | Ctbp2 | -3.06 |
|  |  |  |  |  |
| chr18:53463017-53465407 | 0.53 | - | Prdm6 | 0.93 |
| chr19:45733505-45735997 | 0.53 | DR1 | Fgf8 * | 5.24 |
| chr11:54891361-54894784 | 0.57 | DR1 | Gpx3 | 2.58 |
| chr4:86669294-86671201 | 0.59 | - | Plin2 | 2.29 |
| chr3:127457093-127465482 | 0.62 | - | Ank2 | 2.26 |
| chr18:60492434-60494743 | 0.62 | - | Smim3 | 1.25 |
| chr10:17704835-17706602 | 0.65 | DR1 | Cited2 | 2.13 |
| chr11:57831171-57833707 | 0.66 | - | Hand1 | 1.46 |
| chr6:52310576-52314619 | 0.71 | DR1 | Evx1 | 1.54 |
| chr13:114456392-114460659 | 0.72 | DR2 | Fst * | 1.15 |
| chr5:147298587-147311126 | 0.73 | DR2 | Cdx2 * | 1.98 |
| chr5:53106977-53110254 | 0.74 | DR1 | Sel1l3 | 3.67 |
| chr10:59957002-59959223 | 0.75 | - | Ddit4 | 3.33 |
| chr5:107216257-107218023 | 0.77 | - | Tgfbr3 | 1.37 |
| chr5:104021158-104022631 | 1.000 | - | Hsd17b11 | 3.89 |
| chr1:118647742-118649473 | 1.11 | DR5 | Tfcp2l1 | 2.77 |

ChIP-seq values represent differentially marked H3K27ac peaks comparing *Aldh1a2*-/- (KO) and

wild-type (WT) with BHP <0.05; a cut-off of log2 <-0.51 or >0.51 was employed to include a

differential peak near *Sox2* known to be activated by RA. RNA-seq values represent differentially

expressed genes comparing KO and WT in which FPKM >0.5; a cut-off of log2 <-0.85 or >0.85

was employed to include *Sox2* known to be activated by RA. Genes that have differential peaks for

both H3K27ac and H3K27me3 (Table S2) are marked with an asterisk. RARE, retinoic acid

response element; DR1 or DR2 or DR5, direct repeat with 1 or 2 or 5 bp between each repeat;

TFBS, transcription factor binding site.
