# Supplementary material for: Discovery of genes required for body axis and limb formation by global identification of retinoic acid–regulated epigenetic marks
Source: PLoS Biol. 2020 May 18;18(5):e3000719. doi: 10.1371/journal.pbio.3000719 (PMC7259794; doi:10.1371/journal.pbio.3000719)
Supplement: S2 Table — Aldh1a2, aldehyde dehydrogenase 1A2; ChIP-seq, chromatin immunoprecipitation sequencing; E, embryonic day; H3K27me3, histone H3 K27 trimethylation; RA, retinoic acid. (DOCX) [file pbio.3000719.s002.docx]

S2 Table. Comparison of *Aldh1a2-/-* and wild-type E8.5 trunk tissue for H3K27me3 ChIP-seq and RNA-seq results to identify RA-regulated H3K27me3 ChIP-seq peaks near genes with RA-regulated expression.

| **H3K27me3 ChIP-seq differential peak for *Aldh1a2* KO vs WT**  **(mm10)** | **log2 fold change: H3K27me3 ChIP-seq for *Aldh1a2* KO vs WT** | **RARE: based on Homer TFBS analysis** | **nearby gene with altered expression in *Aldh1a2* KO vs WT** | **log2 fold change for nearby gene: RNA-seq for**  ***Aldh1a2* KO vs WT** |
| --- | --- | --- | --- | --- |
| chr18:38598986-38601292 | -1.20 | - | Spry4 | 3.43 |
| chr17:15533901-15538178 | -0.95 | - | Pdcd2 | 2.22 |
| chr17:15533901-15538178 | -0.95 | - | Tbp | 2.94 |
| chrX:104569467-104572914 | -0.90 | - | Zdhhc15 | 1.5 |
| chrX:104546302-104549188 | -0.89 | - | Zdhhc15 | 1.5 |
| chr2:118901989-118904591 | -0.85 | - | Bahd1 | 1.12 |
| chr4:129226495-129228276 | -0.85 | - | C77080 | 0.98 |
| chr4:129221927-129223300 | -0.77 | - | C77080 | 0.98 |
| chr5:15980910-15984533 | -0.75 | - | Cacna2d1 | 1.01 |
| chr3:89278454-89282169 | -0.72 | - | Efna1 | 1.23 |
| chr11:103110581-103113995 | -0.67 | - | Acbd4 | 6.49 |
| chr4:129246599-129252553 | -0.64 | DR5 | C77080 | 0.98 |
| chr10:21991375-21993681 | -0.64 | - | Sgk1 | 1.38 |
| chr6:125360514-125365732 | -0.63 | DR2 | Tnfrsf1a | 1.39 |
| chr12:54201904-54203715 | -0.63 | - | Egln3 | 3.46 |
| chr5:147297983-147318733 | -0.63 | DR2 | Cdx2 * | 1.98 |
| chrX:104536138-104539780 | -0.62 | DR2 | Zdhhc15 | 1.50 |
| chr4:98726175-98729089 | -0.61 | - | L1td1 | 1.50 |
| chr17:29080591-29082455 | -0.59 | - | Trp53cor1 | 1.91 |
| chr11:117780323-117784425 | -0.58 | DR2 | Tmc8 | 1.33 |
| chr6:122800166-122804076 | -0.54 | - | Slc2a3 | 1.35 |
| chr10:60828600-60835032 | -0.49 | - | Unc5b | 3.58 |
| chr19:45735049-45746658 | -0.49 | DR2 | Fgf8 * | 5.24 |
| chrX:94129800-94133754 | -0.48 | - | Zfx | 1.40 |
| chr2:119235078-119238967 | -0.47 | - | Spint1 | 2.74 |
| chr13:114456076-114460873 | -0.47 | DR2 | Fst * | 1.15 |
| chr19:11816637-11820013 | -0.47 | - | Stx3 | 0.90 |
|  |  |  |  |  |
| chr6:72232803-72239355 | 0.50 | - | Atoh8 | -2.59 |
| chr19:4709301-4715701 | 0.51 | - | Sptbn2 | -1.59 |
| chr3:5219339-5224436 | 0.52 | - | Zfhx4 * | -2.26 |
| chr7:130260543-130263682 | 0.56 | - | Fgfr2 | -3.06 |
| chr17:56471489-56479605 | 0.57 | DR1 | Ptprs * | -3.31 |
| chr4:144893360-144895562 | 0.59 | - | Dhrs3 | -1.11 |
| chr2:116072251-116077455 | 0.61 | DR5 | Meis2 * | -1.10 |
| chr7:70356085-70361002 | 0.63 | DR1 | Nr2f2 * | -2.32 |
| chr15:98621217-98623590 | 0.65 | - | Cacnb3 | -2.73 |
| chr1:59473538-59476300 | 0.66 | DR1 | Fzd7 | -1.64 |
| chr17:47915056-47917877 | 0.67 | - | Foxp4 * | -1.02 |
| chr10:8548515-8549917 | 0.72 | - | Ust | -1.40 |
| chr6:52156115-52158253 | 0.73 | DR5, DR2 | Hoxa1 | -5.43 |
| chr7:96211108-96212622 | 0.75 | - | Tenm4 | -2.65 |
| chrX:162887815-162889313 | 0.76 | - | Syap1 | -1.55 |
| chr11:19015536-19017169 | 0.78 | DR1 | Meis1 * | -2.64 |
| chr18:58208120-58210286 | 0.81 | - | Fbn2 | -1.65 |
| chr14:21983733-21987831 | 0.85 | - | Zfp503 | -2.68 |
| chr11:19007512-19012358 | 0.87 | DR2 | Meis1 * | -2.64 |
| chr14:16574377-16578138 | 1.02 | DR5, DR1 | Rarb * | -1.64 |

ChIP-seq values represent differentially marked H3K27me3 peaks comparing *Aldh1a2*-/- (KO)

and wild-type (WT) with BHP <0.05; a cut-off of log2 <-0.47 or >0.47 was employed to include a

differential peak near *Fst* known to be repressed by RA. RNA-seq values represent differentially

expressed genes comparing KO and WT in which FPKM >0.5; a cut-off of log2 <-0.85 or >0.85

was employed to include the known RA target gene *Sox2*. Genes that have differential peaks for

both H3K27me3 and H3K27ac (Table S1) are marked with an asterisk. RARE, retinoic acid

response element; DR1 or DR2 or DR5, direct repeat with 1 or 2 or 5 bp between each repeat;

TFBS, transcription factor binding site.
