# Supplementary material for: Discovery of genes required for body axis and limb formation by global identification of retinoic acid–regulated epigenetic marks
Source: PLoS Biol. 2020 May 18;18(5):e3000719. doi: 10.1371/journal.pbio.3000719 (PMC7259794; doi:10.1371/journal.pbio.3000719)
Supplement: S3 Table — RAREs contain no more than 2 mismatches to Homer consensus DR5, DR2, or DR1 RARE motifs shown here. ChIP-seq, chromatin immunoprecipitation sequencing; DR, direct repeat; H3K27ac, histone H3 K27 acetylation; H3K27me3, histone H3 K27 trimethylation; RA, retinoic acid; RARE, RA response element; TAD, topologically associated domain. (DOCX) [file pbio.3000719.s003.docx]

S3 Table. DNA sequences of all RAREs located in RA-regulated ChIP-seq peaks for H3K27ac or H3K27me3 near all RA-regulated genes in same TAD. RAREs contain no more than two mismatches to Homer consensus DR5, DR2, or DR1 RARE motifs shown here; DR, direct repeat.

| **RARE MOTIFS:**  **(Homer)** | **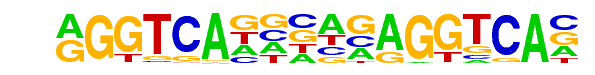**  DR5 = RAR:RXR(NR),DR5 | | **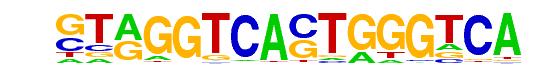** DR2 = Reverb(NR),DR2 | | | | | | | | 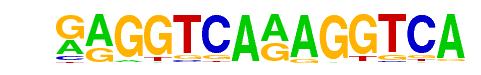 DR1 = TR4(NR),DR1 |
| --- | --- | --- | --- | --- | --- | --- | --- | --- | --- | --- | --- |
| **Nearest gene with decreased or increased expression in *Aldh1a2* KO** | **Other genes in same TAD with decreased or increased expression in *Aldh1a2* KO** | **RARE DNA sequence 5'-3'**  **overall consensus:**  AGGTCA N5,N2 AGGTCA  G T or N1 G T | **Type of RARE** | **Conserved** | | | | | | **Genomic coordinates**  **(mm10)** | |
|  |  |  |  | **rodent** | **human** | **bird** | **reptile** | **frog** | **fish** |  |  |
| **RARE ENHANCERS**  (RA stimulates gain of H3K27ac and/or loss of H3K27me3 near RARE and activates gene in same TAD) | | | | | | | | | | | |
| gene nearest to RARE is RA-activated | gene in same TAD that is RA-activated |  |  |  |  |  |  |  |  |  | |
| Btbd9 | none | AGTTAA C AGGTCA | DR1 | x |  |  |  |  |  | chr17:30471156-30471168 | |
| Cdx1 | Arsi  Dctn4 | GGGTTA G GGGTCA | DR1 | x | x |  |  |  |  | chr18:61036704-61036716 | |
|  |  | GGGTCA AG AGTTCA | DR2 | x | x |  |  |  |  | chr18:61035133-61035146 | |
| Crabp2 | Prcc  Mef2d | AGTTCA CC AGGTCA | DR2 | x |  |  |  |  |  | chr3:87947525-87947538 | |
|  |  | AGGGCA G AGGTCA | DR1 | x | x |  |  |  |  | chr3:87948035-87948047 | |
|  |  | AGGTCA GG AGGGCA | DR2 | x | x |  |  |  |  | chr3:87958618-87958631 | |
| Ctbp2 | Zranb1  Fam53b  Edrf1 | AGGTCT CT GTGTCA | DR2 | - |  |  |  |  |  | chr7:133035195-133035208 | |
|  |  | GGGTCA AT GGGTCT | DR2 | x |  |  |  |  |  | chr7:133037547-133037560 | |
|  |  |  |  |  | | | | | |  | |
| Dach1 | none | AGTTCA CACAA AGTTCA | DR5 | x | x | x | x | x | x | chr14:98035388-98035404 | |
|  |  | GGGACA A AGGTCA | DR1 | x | x |  |  |  |  | chr14:98037394-98037406 | |
| Dhrs3 | none | GGGTCA TTCCA AGTTCA | DR5 | x | x | x | x | x |  | chr4:145034810-145034826 | |
|  |  | GGTTCA TCGGG AGGGCA | DR5 | x | x | x | x | x |  | chr4:145034847-145034863 | |
| Foxp2 | none | AGGTGA A AGTTCA | DR1 | x | x |  |  |  |  | chr6:14898480-14898492 | |
| Foxp4 | none | GGGTGA C AGGTCA | DR1 | x | x | x | x |  |  | chr17:47898625-47898637 | |
| Fzd7 | Tmem237  Stradb | AGGTCA G GGTTCA | DR1 | x | x |  |  |  |  | chr1:59475768-59475780 | |
|  |  |  |  |  |  |  |  |  |  |  | |
| Hoxa1 | Hoxa4  Hoxa9  Skap2 | GGTTCA CCGAA AGTTCA | DR5 | x | x | x | x | x |  | chr6:52153426-52153442 | |
|  |  | AGGTCA CT AAATCA | DR2 | - |  |  |  |  |  | chr6:52156158-52156171 | |
|  |  | GGTTCA AGAAG AGTTCA | DR5 | x | x | x | x | x | x | chr6:52175533-52175549 | |
| Itga9 | none | AGGTCA GCCGG AGGGCA | DR5 | x | x |  |  |  |  | chr9:118655221-118655237 | |
|  |  | AGGCCA A AAGTCA | DR1 | x |  |  |  |  |  | chr9:118656142-118656154 | |
| Lrp8 | none | AGGTCA CT GGGGGA | DR2 | - |  |  |  |  |  | chr4:107836195-107836208 | |
| Meis1 | none | ATGTCA G AGGTCA | DR1 | x | x |  |  |  |  | chr11:19025437-19025449 | |
|  |  | GGGTCA G AGGCCA | DR1 | x | x |  |  |  |  | chr11:19016387-19016399 | |
|  |  | AGGGCA GG GGGCCA | DR2 | x |  |  |  |  |  | chr11:19010468-19010481 | |
|  |  | AGGCCA CTGAG AGGTCA | DR5 | x | x | x | x | x |  | chr11:18963875-18963891 | |
|  |  | ATGTCA A AGGACA | DR1 | x |  |  |  |  |  | chr11:18958299-18958311 | |
| Meis2 | Dph6 | AGGTCA AAAAC AGTTCA | DR5 | x | x | x | x |  |  | chr2:116071242-116071258 | |
|  |  | CTCTCA AA GGGTCA | DR2 | x | x |  |  |  |  | chr2:116020707-116020720 | |
| Mtcl1 | none | GGGTCA GGAGG AGTTGA | DR5 | - |  |  |  |  |  | chr17:66412903-66412919 | |
|  |  | GGGTCA C AGGTCA | DR1 | x | x |  |  |  |  | chr17:66413174-66413186 | |
| Nr2f1 | none | GTGTCA A AGTTCA | DR1 | x | x | x | x | x | x | chr13:78200425-78200437 | |
| Nr2f2 | none | GTGTCA A AGTTCA | DR1 | x | x | x | x | x | x | chr7:70361772-70361784 | |
| Ppp1r9a | Casd1  Gng11 | GGGTCA AGGGC ATATCA | DR5 | - |  |  |  |  |  | chr6:5030269-5030285 | |
|  |  | AGGGCA CT GGCTCA | DR2 | - |  |  |  |  |  | chr6:5031044-5031057 | |
| Ptprs | Zfp119a  Ranbp3  Ndufa11 | GGGTCA CG AGGTCA | DR2 | x |  |  |  |  |  | chr17:56470658-56470671 | |
|  |  | AGGTCA CC AGGTCA | DR2 | x |  |  |  |  |  | chr17:56470666-56470679 | |
|  |  | AGGTCA C AGGGCA | DR1 | - |  |  |  |  |  | chr17:56470674-56470686 | |
|  |  | GGATCA GG AGTTCA | DR2 | x |  |  |  |  |  | chr17:56471720-56471733 | |
| Rarb | none | GGTTCA CCGAA AGTTCA | DR5 | x | x | x | x |  |  | chr14:16575513-16575529 | |
|  |  | AGGACA G AGGTCA | DR1 | - |  |  |  |  |  | chr14:16578037-16578049 | |
| Robo2 | none | GTGGCA A AGGTCA | DR1 | - |  |  |  |  |  | chr16:74398869-74398881 | |
| Smad3 | none | GGGTCA TGTGA AGTTCA | DR5 | x | x |  |  |  |  | chr9:63716397-63716413 | |
|  |  |  |  |  |  |  |  |  |  |  | |
| Sox2 | none | GGGTCA GG AGGTCA | DR2 | x | x | x | x | x | x | chr3:34679067-34679080 | |
| Spsb4 | none | GGGTCA CGCAC GGGTCA | DR5 | x |  |  |  |  |  | chr9:96989808-96989824 | |
|  |  | AGCTCA CT GGGGCA | DR2 | - |  |  |  |  |  | chr9:96958298-96958311 | |
| Stx8 | Ntn1 | AGTTCA G AGTTCA | DR1 | x |  |  |  |  |  | chr11:68051350-68051362 | |
|  |  | AGTTCA CT GTGGCA | DR2 | x | x |  |  |  |  | chr11:68051567-68051580 | |
| Tshz1 | none | GGGTCA TTCAT AGTTCA | DR5 | x | x | x | x |  |  | chr18:84073476-84073492 | |
|  |  | AGGTCA CCCAG AGTTCA | DR5 | x |  |  |  |  |  | chr18:84075146-84075162 | |
|  |  | AGGTCA GG AGGTGA | DR2 | x | x | x | x |  |  | chr18:83839858-83839871 | |
|  |  | GGGTGA ACTCA GGTTCA | DR5 | x | x | x | x |  |  | chr18:83839869-83839885 | |
| Ttc28 | Chek2 | AGGTCA G AGGTTA | DR1 | x |  |  |  |  |  | chr5:111244585-111244597 | |
| Tubb2b | Psmg4 | GTGTCA GT GGGTCT | DR2 | x |  |  |  |  |  | chr13:34130267-34130280 | |
| Vwa8 | none | GAGTCA A AGGTCA | DR1 | x |  |  |  |  |  | chr14:78849683-78849695 | |
|  |  | AGGTCA TACAC AGGCCA | DR5 | - |  |  |  |  |  | chr14:78850773-78850789 | |
|  |  | GGCTTA CT GGGTCA | DR2 | - |  |  |  |  |  | chr14:78851047-78851060 | |
|  |  | GGGTCA A AGTTCA | DR1 | x |  |  |  |  |  | chr14:78851055-78851067 | |
| Zbtb16 | none | GGGTCA CA GGGTCA | DR2 | x | x | x | x | - | x | chr9:48694721-48694734 | |
|  |  | GGGTCA G GGGTTA | DR1 | x | x | x | x |  |  | chr9:48695827-48695839 | |
|  |  | GGGTCA G AGGCCA | DR1 | x | x |  |  |  |  | chr9:48696900-48696912 | |
| Zfand5 | none | GGGTCA TT GGGTAA | DR2 | x |  |  |  |  |  | chr19:21165208-21165221 | |
| Zfhx4 | Pex2 | GGGTCA GCCTG AGGTCA | DR5 | x | x | x | x | x | x | chr3:5388103-5388119 | |
| Zfp638 | none | GGTTCA GCCAA AGGTGA | DR5 | x | x | x | x | x |  | chr6:84976840-84976856 | |
| 1700017B05Rik | Commd4  Man2c1 | AGGTAA A AGGTCA | DR1 | x | x |  |  |  |  | chr9:57265274-57265286 | |
|  |  | GGGTCT CT GGGTCT | DR2 | - |  |  |  |  |  | chr9:57266886-57266899 | |
|  |  | 65 RARE enhancers where nearest gene is RA-activated |  |  |  |  |  |  |  |  | |
| (gene nearest to RARE is not RA-activated) | gene in same TAD that is RA-activated |  |  |  |  |  |  |  |  |  | |
| (Gm15428) | Ralgps2  Fam20b | GGGTCA G AGATCA | DR1 | x |  |  |  |  |  | chr1:156789360-156789372 | |
|  |  | GGGTCA GTGAG GGGTCA | DR5 | x |  |  |  |  |  | chr1:156790306-156790322 | |
| (Gm37839) | Lmx1a  Pbx1 | GGGTCA AACGC AGGGCA | DR5 | x | x |  |  |  |  | chr1:169238665-169238681 | |
|  |  | GGGTCG CT GGGTCA | DR2 | x | x | x | x |  |  | chr1:169238844-169238857 | |
| (Gm6075) | Crispld1 | ATGTCA GT AGGACA | DR2 | - |  |  |  |  |  | chr1:17450456-17450469 | |
| (Gm37068) | Ccdc115 | AGGTCA TTCAA AGGTCA | DR5 | x |  |  |  |  |  | chr1:35694541-35694557 | |
| (Col4a4) | Irs1 | AGGTCA A AGGTCA | DR1 | - |  |  |  |  |  | chr1:82475916-82475928 | |
| (Gm28884) | B3gnt7 | GGGTCA GACAC AGGGGA | DR5 | - |  |  |  |  |  | chr1:85933157-85933173 | |
| (Acoxl) | Bcl2l11 | GGGTCA G AGGCCA | DR1 | x | x |  |  |  |  | chr2:127907834-127907846 | |
| (1700001O22Rik)  (Ptges) | Prrx2  Ntmt1 | AGTTCA A GGTTAT | DR1 | - |  |  |  |  |  | chr2:30779109-30779121 | |
|  |  | AGGCCA GGCAG AGGTCA | DR5 | x | x |  |  |  |  | chr2:30779417-30779433 | |
|  |  | GGGTCA CAGAG AGGTCA | DR5 | x | x |  |  |  |  | chr2:30779575-30779591 | |
|  |  | GGGTCA G AGGCGA | DR1 | x |  |  |  |  |  | chr2:30893114-30893126 | |
|  |  | AGTTCA A AGTTGA | DR1 | x |  |  |  |  |  | chr2:30893953-30893965 | |
|  |  | AGTTCA AGGTC AGTGCT | DR5 | x |  |  |  |  |  | chr2:30894156-30894172 | |
| (Gm25869) | Olfml3 | AGGTCA GGGAG AAGTCA | DR5 | x |  |  |  |  |  | chr3:103419827-103419843 | |
|  |  | AGGTCA AGGAG GATTCT | DR5 | x | x |  |  |  |  | chr3:103420712-103420728 | |
| (Mcoln2) | Prkacb | AGGTCA C AGGTCA | DR1 | x |  |  |  |  |  | chr3:146204096-146204108 | |
|  |  | GGGTCA CACAG GGGTCA | DR5 | x |  |  |  |  |  | chr3:146204537-146204553 | |
| (Gm37359) | Tiparp | AGGTCA CA GGGTCA | DR2 | x |  |  |  |  |  | chr3:65859155-65859168 | |
| (Trim62) | Zscan20 | GGGTCA CA GGGTCA | DR2 | x |  |  |  |  |  | chr4:128897746-128897759 | |
|  |  | AGGTCT GG GGGGCA | DR2 | - |  |  |  |  |  | chr4:128898695-128898708 | |
| (Gm12992) | Ythdf2 | AGGTCA CACAG AGGCCA | DR5 | x | x |  |  |  |  | chr4:131918909-131918925 | |
|  |  | GGGCCA G AGTTCA | DR1 | x |  |  |  |  |  | chr4:131919741-131919753 | |
| (Grrp1) | Pdik1l  Dhdds | AGGTGG G AGGTCA | DR1 | x |  |  |  |  |  | chr4:134256564-134256576 | |
|  |  | AGTTGA G AGGTGA | DR1 | - |  |  |  |  |  | chr4:134257704-134257716 | |
| (Gm13200) | Mad2l2 | GGGGCA AGCAG GGGTCA | DR5 | x |  |  |  |  |  | chr4:148383603-148383619 | |
|  |  | AAGTCA CC GGGTCA | DR2 | - |  |  |  |  |  | chr4:148383993-148384006 | |
| (Spsb1) | Clstn1  Lzic  Nmnat1  Kif1b | GGGTCA GA AGGTCA | DR2 | x | x | x | x | x |  | chr4:149907094-149907107 | |
|  |  | AGGTCA G AGGGCA | DR1 | x |  |  |  |  |  | chr4:150095743-150095755 | |
|  |  |  |  |  |  |  |  |  |  |  | |
| (Whrn) | Akna | GGGTCA CG GGGTCG | DR2 | - |  |  |  |  |  | chr4:63466825-63466838 | |
| (Stx2) | Fzd10  Mmp17  Snora15  Sumf2  Chchd2  Gusb | AGGTCA G AGGGAA | DR1 | - |  |  |  |  |  | chr5:128975872-128975884 | |
|  |  | AGGTCA TCCTG AGGGCA | DR5 | x |  |  |  |  |  | chr5:128976529-128976545 | |
|  |  |  |  |  |  |  |  |  |  |  | |
| (Clip2) | Abhd11  Bcl7b | GGGTCA CCGAG AGGTCA | DR5 | x | x |  |  |  |  | chr5:134541736-134541752 | |
|  |  | AGGTTA T AGGTCA | DR1 | - |  |  |  |  |  | chr5:134542981-134542993 | |
| (Wasf3) | Rnf6 | GGGTGG G AGGTCA | DR1 | - |  |  |  |  |  | chr5:146386603-146386615 | |
| (Uspl1) | Ubl3 | AGGTCA A AGGTCA | DR1 | - |  |  |  |  |  | chr5:149196756-149196768 | |
|  |  | GGGTCA AACTC AGGTCA | DR5 | x |  |  |  |  |  | chr5:149196825-149196841 | |
| (Dgki) | Ptn | GGGTCA GGGTG AGCACA | DR5 | x |  |  |  |  |  | chr6:36880813-36880829 | |
| (8030453O22Rik) | Ndufb2 | AGTTCA GT GGCTCA | DR2 | - |  |  |  |  |  | chr6:39545227-39545240 | |
| (Gm5876) | Prdm5 | AGGTCA GCAGC AGGTCA | DR5 | x | x |  |  |  |  | chr6:66077462-66077478 | |
| (Efcc1) | Isy1 | AGGTCA G AGGTCA | DR1 | x |  |  |  |  |  | chr6:87739484-87739496 | |
| (Gm23847) | Fgfr2  Ate1 | GGTTCA G AGTTCA | DR1 | x |  |  |  |  |  | chr7:130009273-130009285 | |
|  |  |  |  |  |  |  |  |  |  |  | |
| (Nav2) | Prmt3 | AGGTCA TAAAC AAGTCA | DR5 | x |  |  |  |  |  | chr7:49333913-49333929 | |
| (Gse1) | Tldc1  Hsdl1  Zdhhc7  Gins2 | GGGTCA A AGGGGA | DR1 | - |  |  |  |  |  | chr8:120408671-120408683 | |
|  |  | AGGGCA G AGGGCA | DR1 | - |  |  |  |  |  | chr8:120409479-120409491 | |
|  |  |  |  |  |  |  |  |  |  |  | |
| (Igdcc3) | Dpp8 | GAGTCA A AGGGCA | DR1 | x | x |  |  |  |  | chr9:65163164-65163176 | |
| (E130307A14Rik) | Rpf2 | GGGTCA A AGGTCA | DR1 | x | x |  |  |  |  | chr10:39660884-39660896 | |
| (Polr3b) | Ric8b | GTTTCA A AGGTCA | DR1 | - |  |  |  |  |  | chr10:84710728-84710740 | |
| (Evpl) | Unk  Mrpl38  Srp68 | GGATCA GA AGTTCA | DR2 | x |  |  |  |  |  | chr11:116241241-116241254 | |
|  |  |  |  |  |  |  |  |  |  |  | |
| (Gm28401) | C1d | GGGTCA G GGGTTA | DR1 | x | x | x | x | x | x | chr11:18748180-18748192 | |
|  |  | GGATCA GT GTGTCA | DR2 | x | x |  |  |  |  | chr11:18749733-18749746 | |
| (Cdk5rap3)  (Gm11523) | Scrn2  Hoxb3  Hoxb5os | GGGTCA G AGGGCA | DR1 | - |  |  |  |  |  | chr11:96896439-96896451 | |
|  |  | AGGTCA G AGGTGA | DR1 | - |  |  |  |  |  | chr11:96897098-96897110 | |
|  |  | AGGACA G AGGTCA | DR1 | - | x |  |  |  |  | chr11:96899490-96899502 | |
|  |  | GGGTCA G GGGGAGA | DR1 | - |  |  |  |  |  | chr11:96899889-96899902 | |
|  |  | GACTCA AG AGTTCA | DR2 | - |  |  |  |  |  | chr11:96877903-96877916 | |
| (Fam181a) | Otub2 | GGTTAC TG AGGTCA | DR2 | x | x |  |  |  |  | chr12:103314689-103314702 | |
| (Gm5441) | Zfp386 | GAGTCA A AGGTCA | DR1 | x | - | x | x |  |  | chr12:117352086-117352098 | |
| (Gm25538)  (Ddx1) | Fam49a  Mycn | AGGTCA A AGGTGA | DR1 | x | x |  |  |  |  | chr12:12723995-12724007 | |
|  |  | GGGTGA A AGGTCA | DR1 | x |  |  |  |  |  | chr12:13207084-13207096 | |
|  |  | AGTTCA A GGTCCT | DR1 | x |  |  |  |  |  | chr12:13209724-13209736 | |
| (Agr3) | Ispd | GGGGCA ATGTG AGGTCA | DR5 | x |  |  |  |  |  | chr12:35894512-35894528 | |
|  |  | AGTTCA G GGGTCA | DR1 | x | x |  |  |  |  | chr12:35894569-35894581 | |
| (Unc5a) | Rab24 | GGGTTA G AGTTCA | DR1 | x | x |  |  |  |  | chr13:54993593-54993605 | |
|  |  | GGGTCA CT GGCTTA | DR2 | x | x |  |  |  |  | chr13:54994828-54994841 | |
| (B230219D22Rik) | Grk6  Ddx41 | AGGTCA TA GGGTCA | DR2 | x |  |  |  |  |  | chr13:55688082-55688095 | |
|  |  |  |  |  |  |  |  |  |  |  | |
| (Kif13b) | Ints9  Fzd3 | GGGTCA AACTC AGGTCA | DR5 | x |  |  |  |  |  | chr14:64640716-64640732 | |
|  |  |  |  |  |  |  |  |  |  |  | |
| (Snora31) | Afap1  Trmt44 | GGGTCA GTCAG GGGTCA | DR5 | x |  |  |  |  |  | chr14:75863890-75863906 | |
|  |  |  |  |  |  |  |  |  |  |  | |
| (Celsr1) | Cerk  Trmu | AGGTCA GA GGCTCA | DR2 | x |  |  |  |  |  | chr15:85971844-85971857 | |
|  |  |  |  |  |  |  |  |  |  |  | |
| (Gm15742) | Ppp1r2 | AGTTGA A AGGCCA | DR1 | x |  |  |  |  |  | chr16:30939753-30939765 | |
| (Spice1) | Naa50 | AGGTCA CA GTGTGA | DR2 | x | x |  |  |  |  | chr16:44382205-44382218 | |
|  |  | ATTTCT G AGTTCA | DR1 | - |  |  |  |  |  | chr16:44382403-44382415 | |
| (Gm26694) | Capn15  Wdr24  Fbxl16  Wdr90  Ube2i  Ift140  Nme3 | AGGTCA CCACC AGGCCA | DR5 | x | x |  |  |  |  | chr17:25795888-25795904 | |
|  |  |  |  |  |  |  |  |  |  |  | |
| (Gm26682) | Zeb1 | AGGTCA ACCAG AGGGCA | DR5 | x | x |  |  |  |  | chr18:5186998-5187014 | |
| (Gm14505) | Cask | GGTTCA CAGAA AGTTCA | DR5 | x |  |  |  |  |  | chrX:14628834-14628850 | |
| (Actrt3) | Skil | GAGGCA GG AGGTCA | DR2 | - |  |  |  |  |  | chr3:30596235-30596248 | |
|  |  | GAGTCA CT GGGTCA | DR2 | - |  |  |  |  |  | chr3:30596543-30596556 | |
| (Slfn14) | Nle1  Zfp830 | AGGGCA CAGCA AGGTCA | DR5 | x |  |  |  |  |  | chr11:83277949-83277965 | |
|  |  |  |  |  |  |  |  |  |  |  | |
| (Sacs) | Tnfrsf19  Mipep | AGGTCA GA GGGAGA | DR2 | - |  |  |  |  |  | chr14:61167047-61167060 | |
|  |  |  |  |  |  |  |  |  |  |  | |
| (Sp7) | Aaas  Pfdn5 | AGGTGA GCTTG AGGCCA | DR5 | x |  |  |  |  |  | chr15:102365172-102365188 | |
|  |  | GGGTCA G AGGGCA | DR1 | x | x |  |  |  |  | chr15:102366013-102366025 | |
|  |  | 82 RARE enhancers where nearest gene is not RA-activated but other gene(s) in TAD are |  |  |  |  |  |  |  |  | |
| **RARE SILENCERS**  (RA stimulates gain of H3K27me3 and/or loss of H3K27ac near RARE and represses gene in same TAD) | | | | | | | | | | | |
| gene nearest to RARE is RA-repressed | gene in same TAD that is RA-repressed |  |  |  |  |  |  |  |  |  | |
| C77080 | Zbtb8os | GGGTGA TCCAA AGGCCA | DR5 | - |  |  |  |  |  | chr4:129248733-129248749 | |
| Cdx2 | Lnx2  Usp12 | GGGTCA CT GGGTGA | DR2 | x | x |  |  |  |  | chr5:147301839-147301852 | |
|  |  | GGCTCA CA GTGTCA | DR2 | - |  |  |  |  |  | chr5:147302661-147302674 | |
|  |  | AGGTCA CT TGGTCA | DR2 | x | x |  |  |  |  | chr5:147303936-147303949 | |
| Cited2 | Heca | AGATGA G AGGTCA | DR1 | - |  |  |  |  |  | chr10:17705156-17705168 | |
| Evx1 | none | GGGGCA G AGGTGA | DR1 | - |  |  |  |  |  | chr6:52312241-52312253 | |
| Fgf8 | Poll  Btrc  Mrpl43  Sema4g  Chuk  Erlin1  Dnmbp  Entpd7  Got1  Slc25a28 | GGGTCA GC AGTTCA | DR2 | x | x | x |  |  |  | chr19:45747043-45747056 | |
|  |  | AGGTCT CT GGGTCG | DR2 | - |  |  |  |  |  | chr19:45743342-45743355 | |
|  |  | AGGGCA G AGGCCA | DR1 | x |  |  |  |  |  | chr19:45735030-45735042 | |
|  |  |  |  |  |  |  |  |  |  |  | |
| Fst | Ndufs4  Mocs2 | GGGGCA GG GGTTCT | DR2 | x |  |  |  |  |  | chr13:114458455-114458468 | |
|  |  |  |  |  |  |  |  |  |  |  | |
| Gpx3 | none | GGATCA A AGTTCA | DR1 | x |  |  |  |  |  | chr11:54892620-54892632 | |
|  |  | GGGTCA G AGGTCG | DR1 | - |  |  |  |  |  | chr11:54892779-54892791 | |
| Sel1l3 | Slc34a2 | AGGTCA G AGGTCA | DR1 | x |  |  |  |  |  | chr5:53109896-53109908 | |
|  |  | GAGTCA A AGTTCA | DR1 | x | x |  |  |  |  | chr7:27356622-27356634 | |
| Tfcp2l1 | none | AGGTCA TTATC AGGTGA | DR5 | x |  |  |  |  |  | chr1:118648735-118648751 | |
| Tmc8 | Syngr2  Tk1 | TGGTCA GT GGGTCT | DR2 | x |  |  |  |  |  | chr11:117782965-117782978 | |
|  |  | GGGTCA TG GGGACA | DR2 | x |  |  |  |  |  | chr11:117784333-117784346 | |
| Tnfrsf1a | Cd9  Nop2  Spsb2  Ptpn6 | AGGTCA TG GAGTCA | DR2 | x |  |  |  |  |  | chr6:125360617-125360630 | |
|  |  |  |  |  |  |  |  |  |  |  | |
| Zdhhc15 | none | AGGTCT GT GGGCCA | DR2 | - |  |  |  |  |  | chrX:104539271-104539284 | |
|  |  | GGGTCC CTGTG AGTTCA | DR5 | - |  |  |  |  |  | chrX:104571199-104571215 | |
|  |  | 20 RARE silencers where nearest gene is RA-repressed |  |  |  |  |  |  |  |  | |
| nearest gene not RA-repressed | gene in same TAD that is RA-repressed |  |  |  |  |  |  |  |  |  | |
| (Gm13686) | Tfpi | GGGTCA A AGGTGA | DR1 | x |  |  |  |  |  | chr2:83709948-83709960 | |
| (Accsl) | Cd82  Tspan18 | AGGGCA A AGGTCA | DR1 | x |  |  |  |  |  | chr2:93873962-93873974 | |
|  |  |  |  |  |  |  |  |  |  |  | |
| (Wnt5b) | Erc1  Adipor2 | AGGTCA AG GGCTCA | DR2 | - |  |  |  |  |  | chr6:119448467-119448480 | |
|  |  |  |  |  |  |  |  |  |  |  | |
| (Lrrc27) | Pwwp2b | AGTTCA A AGTCCA | DR1 | x |  |  |  |  |  | chr7:139234821-139234833 | |
| (Ldha) | Hps5 | GAATCA G AGTTCA | DR1 | x |  |  |  |  |  | chr7:46837621-46837633 | |
|  |  | AGCTCA CT AGGCCA | DR2 | x | x |  |  |  |  | chr7:46839662-46839675 | |
|  |  | AGGGCA A AGGTGA | DR1 | x | x |  |  |  |  | chr7:46839730-46839742 | |
| (Arid5b) | Rhobtb1 | AGGTCA GAGAA AGGTCA | DR5 | x |  |  |  |  |  | chr10:68229857-68229873 | |
| (C030005K06Rik) | Dtwd2 | AGGGCA A AGGGCA | DR1 | - |  |  |  |  |  | chr18:50052943-50052955 | |
| (Epha2) | Plekhm2  Dnajc16 | GAGGCA G AGGTCA | DR1 | x |  |  |  |  |  | chr4:141300510-141300522 | |
|  |  |  |  |  |  |  |  |  |  |  | |
| (Hpse) | Plac8 | GGATCA GC AGTTCA | DR2 | x |  |  |  |  |  | chr5:100717951-100717964 | |
|  |  | AGATCA AA AGTTCA | DR2 | x |  |  |  |  |  | chr5:100718336-100718349 | |
|  |  | AGGTCA GT GGGACA | DR2 | - |  |  |  |  |  | chr5:100718666-100718679 | |
| (Sh2b3) | Trafd1 | AGGTCA G GGTCAA | DR1 | - |  |  |  |  |  | chr5:121839252-121839264 | |
| (Cux1) | Znhit1 | AGGCTA TG AGTTCA | DR2 | - |  |  |  |  |  | chr5:136582974-136582987 | |
| (4933427G23Rik) | Rint1 | ACGTCA GT GGGACA | DR2 | - |  |  |  |  |  | chr5:23830755-23830768 | |
| (Shkbp1) | Numbl  Sertad1  Akt2  Zfp60  Rps16  Dll3  Paf1  Fbxo17 | GAGTCA A AGTTCA | DR1 | x | x |  |  |  |  | chr7:27356622-27356634 | |
|  |  | GGATTA TATTG AGTTCA | DR5 | x |  |  |  |  |  | chr7:28377231-28377247 | |
|  |  |  |  |  |  |  |  |  |  |  | |
| (Usp10) | Cotl1  Taf1c  Necab2 | AGGGCA GG AGGACA | DR2 | x |  |  |  |  |  | chr8:119898220-119898233 | |
|  |  |  |  |  |  |  |  |  |  |  | |
| (Gm4895) | Sgk1 | AGGCCA G AAGTCA | DR1 | - |  |  |  |  |  | chr10:22156107-22156119 | |
|  |  | AGGGAA A AGGTCA | DR1 | - |  |  |  |  |  | chr10:22160643-22160655 | |
| (Rnf213) | Sgsh  Gaa  Ccdc40  Chmp6 | GGGGCA G AGGCCA | DR1 | - |  |  |  |  |  | chr11:119392214-119392226 | |
|  |  | AGTCCA GGCCA AGGACA | DR5 | - |  |  |  |  |  | chr11:119392289-119392305 | |
|  |  | AGGTCA TAGGT AGTCCA | DR5 | x |  |  |  |  |  | chr11:119392300-119392316 | |
|  |  |  |  |  |  |  |  |  |  |  | |
| (Camk2b) | Ykt6  Ddx56  Zmiz2 | GGGTCA T AGGCCA | DR1 | - |  |  |  |  |  | chr11:6007158-6007170 | |
|  |  |  |  |  |  |  |  |  |  |  | |
| (Gm16505) | Asb13  Klf6 | AGTCCA A AGGTCA | DR1 | - |  |  |  |  |  | chr13:3362568-3362580 | |
|  |  |  |  |  |  |  |  |  |  |  | |
| (Il17rd) | Arhgef3  Fam208a | TGGTCA A AGGGCA | DR1 | - |  |  |  |  |  | chr14:27039866-27039878 | |
|  |  |  |  |  |  |  |  |  |  |  | |
| (Gm7030)  (2410017I17Rik) | Ddr1  Flot1  Prr3 | GTGTCA G AGGTCA | DR1 | x |  |  |  |  |  | chr17:36128857-36128869 | |
|  |  | AGGCCA G AGGTCA | DR1 | - |  |  |  |  |  | chr17:36155723-36155735 | |
|  |  | GGGTCG G AGGTCA | DR1 | x |  |  |  |  |  | chr17:36167901-36167913 | |
| (4930519F16Rik) | Cdx4  Chic1 | ATTTCT G AGTTCA | DR1 | - |  |  |  |  |  | chrX:103254783-103254795 | |
|  |  | AGTTCA G AGGTTA | DR1 | x |  |  |  |  |  | chrX:103256648-103256660 | |
|  |  | AGGCCA G AGGGCA | DR1 | x |  |  |  |  |  | chrX:103257644-103257656 | |
|  |  | ATTTCT G AGTTCA | DR1 | - |  |  |  |  |  | chrX:103285450-103285462 | |
|  |  | ATTTCT G AGTTCA | DR1 | - |  |  |  |  |  | chrX:103337616-103337628 | |
|  |  | ATTTCT G AGTTCA | DR1 | - |  |  |  |  |  | chrX:103339320-103339332 | |
|  |  | ATTTCT G AGTTCA | DR1 | - |  |  |  |  |  | chrX:103342427-103342439 | |
|  |  | ATTTCT G AGTTCA | DR1 | - |  |  |  |  |  | chrX:103344477-103344489 | |
| (Gm15232) | Rab9 | AAGTCA G AGGTCA | DR1 | x |  |  |  |  |  | chrX:167125774-167125786 | |
|  |  | AGGCCA G AGGTCA | DR1 | x |  |  |  |  |  | chrX:167125965-167125977 | |
|  |  | 40 RARE silencers where nearest gene is not RA-repressed but other gene(s) in TAD are |  |  |  |  |  |  |  |  | |
