# Supplementary material for: Discovery of genes required for body axis and limb formation by global identification of retinoic acid–regulated epigenetic marks
Source: PLoS Biol. 2020 May 18;18(5):e3000719. doi: 10.1371/journal.pbio.3000719 (PMC7259794; doi:10.1371/journal.pbio.3000719)
Supplement: S4 Table — ChIP-seq, chromatin immunoprecipitation sequencing; EB, embryoid body; panRAR, including all RA receptor subtypes; RARE, retinoic acid response element. (DOC) [file pbio.3000719.s004.doc]

S4 Table. DNA sequences of highly conserved RAREs and relationship to panRAR ChIP-seq

peaks for mouse embryoid bodies (EB) (Moutier et al., 2012) and F9 embryonal carcinoma cells (Chatagnon

et al., 2015) reported using mm9 genomic coordinates.

| **Nearest gene with decreased or increased expression in *Aldh1a2* KO** | **RARE DNA sequence 5'-3'**  **overall consensus:**  AGGTCA N5,N2 AGGTCA  G T or N1 G T | **Genomic coordinates**  **(mm10)** | **Genomic coordinates**  **(mm9)** | **Located in RAR ChIP-seq peak** | |
| --- | --- | --- | --- | --- | --- |
|  |  |  |  | EB | F9 |
| RARE enhancers |  |  |  |  |  |
| C1d | GGGTCA G GGGTTA | chr11:18748180-18748192 | chr11:18648183-18648195 | yes | yes |
| Clstn1 | GGGTCA GA AGGTCA | chr4:149907094-149907107 | chr4:149281203-149281216 | no | yes |
| Dach1 | AGTTCA CACAA AGTTCA | chr14:98035388-98035404 | chr14:98434607-98434623 | no | no |
| Dhrs3 | GGGTCA TTCCA AGTTCA | chr4:145034810-145034826 | chr4:144624713-144624729 | no | yes |
|  | GGTTCA TCGGG AGGGCA | chr4:145034847-145034863 | chr4:144624750-144624766 | no | yes |
| Foxp4 | GGGTGA C AGGTCA | chr17:47898625-47898637 | chr17:48035574-48035586 | yes | yes |
| Hoxa1 | GGTTCA CCGAA AGTTCA | chr6:52153426-52153442 | chr6:52103425-52103441 | yes | yes |
|  | GGTTCA AGAAG AGTTCA | chr6:52175533-52175549 | chr6:52125532-52125548 | no | yes |
| Meis1 | AGGCCA CTGAG AGGTCA | chr11:18963875-18963891 | chr11:18863878-18863894 | yes | no |
| Meis2 | AGGTCA AAAAC AGTTCA | chr2:116071242-116071258 | chr2:115896978-115896994 | yes | no |
| Nr2f1 | GTGTCA A AGTTCA | chr13:78200425-78200437 | chr13:78339686-78339698 | yes | no |
| Nr2f2 | GTGTCA A AGTTCA | chr7:70361772-70361784 | chr7:77506658-77506670 | yes | no |
| Pbx1 | GGGTCG CT GGGTCA | chr1:169238844-169238857 | chr1:171168975-171168988 | yes | no |
| Rarb | GGTTCA CCGAA AGTTCA | chr14:16575513-16575529 | chr14:17408027-17408043 | yes | yes |
| Sox2 | GGGTCA GG AGGTCA | chr3:34679067-34679080 | chr3:34577989-34578002 | yes | yes |
| Tshz1 | GGGTCA TTCAT AGTTCA | chr18:84073476-84073492 | chr18:84242868-84242884 | no | no |
|  | AGGTCA GG AGGTGA | chr18:83839858-83839871 | chr18:84009250-84009263 | yes | yes |
|  | GGGTGA ACTCA GGTTCA | chr18:83839869-83839885 | chr18:84009261-84009277 | yes | yes |
| Zbtb16 | GGGTCA CA GGGTCA | chr9:48694721-48694734 | chr9:48502826-48502839 | no | no |
|  | GGGTCA G GGGTTA | chr9:48695827-48695839 | chr9:48503932-48503944 | no | no |
| Zfhx4 | GGGTCA GCCTG AGGTCA | chr3:5388103-5388119 | chr3:5388103-5388119 | yes | no |
| Zfp386 | GAGTCA A AGGTCA | chr12:117352086-117352098 | chr12:118590559-118590571 | yes | yes |
| Zfp638 | GGTTCA GCCAA AGGTGA | chr6:84976840-84976856 | chr6:84926834-84926850 | no | no |
|  |  |  |  |  |  |
| RARE  silencers |  |  |  |  |  |
| Fgf8 | GGGTCA GC AGTTCA | chr19:45747043-45747056 | chr19:45821533-45821546 | yes | yes |
