# Supplementary figures and images for: Discovery of genes required for body axis and limb formation by global identification of retinoic acid–regulated epigenetic marks
Source: PLoS Biol. 2020 May 18;18(5):e3000719. doi: 10.1371/journal.pbio.3000719 (PMC7259794; doi:10.1371/journal.pbio.3000719)

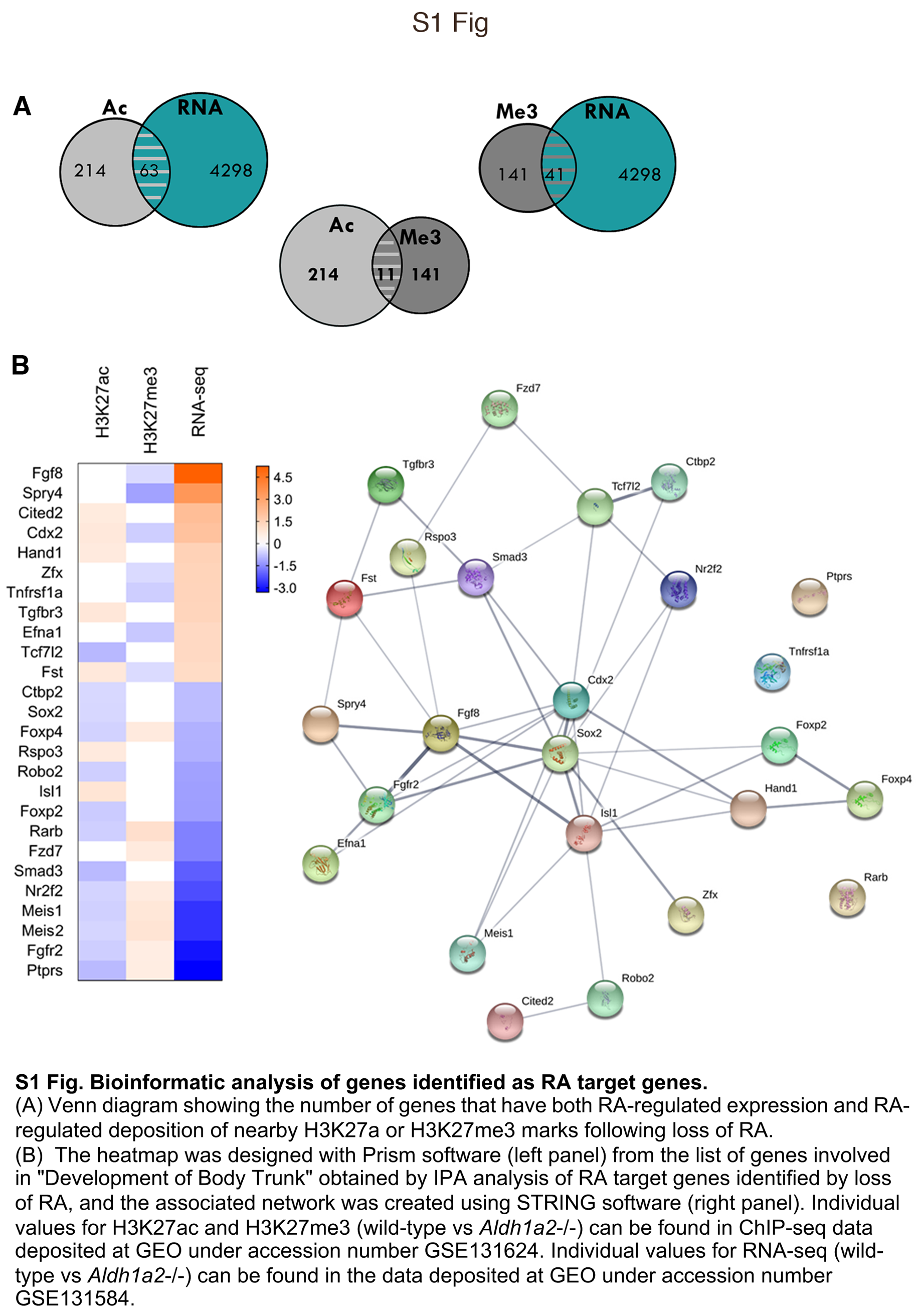

Supplement: S1 Fig — (A) Venn diagram showing the number of genes that have both RA-regulated expression and RA-regulated deposition of nearby H3K27a or H3K27me3 marks following loss of RA. (B) The heatmap was designed with Prism software (left panel) from the list of genes involved in "Development of Body Trunk" obtained by IPA analysis of RA target genes identified by loss of RA, and the associated network was created using STRING software (right panel). Individual values for H3K27ac and H3K27me3 (wild type versus Aldh1a2-/-) can be found in ChIP-seq data deposited at GEO under accession number GSE131624. Individual values for RNA-seq (wild type versus Aldh1a2-/-) can be found in the data deposited at GEO under accession number GSE131584. Aldh1a2, aldehyde dehydrogenase 1A2; ChIP-seq, chromatin immunoprecipitation sequencing; GEO, Gene Expression Omnibus; H3K27ac, histone H3 K27 acetylation; H3K27me3, histone H3 K27 trimethylation; IPA, Ingenuity Pathway Analysis; RA, retinoic acid. (TIF) [file pbio.3000719.s005.tif]

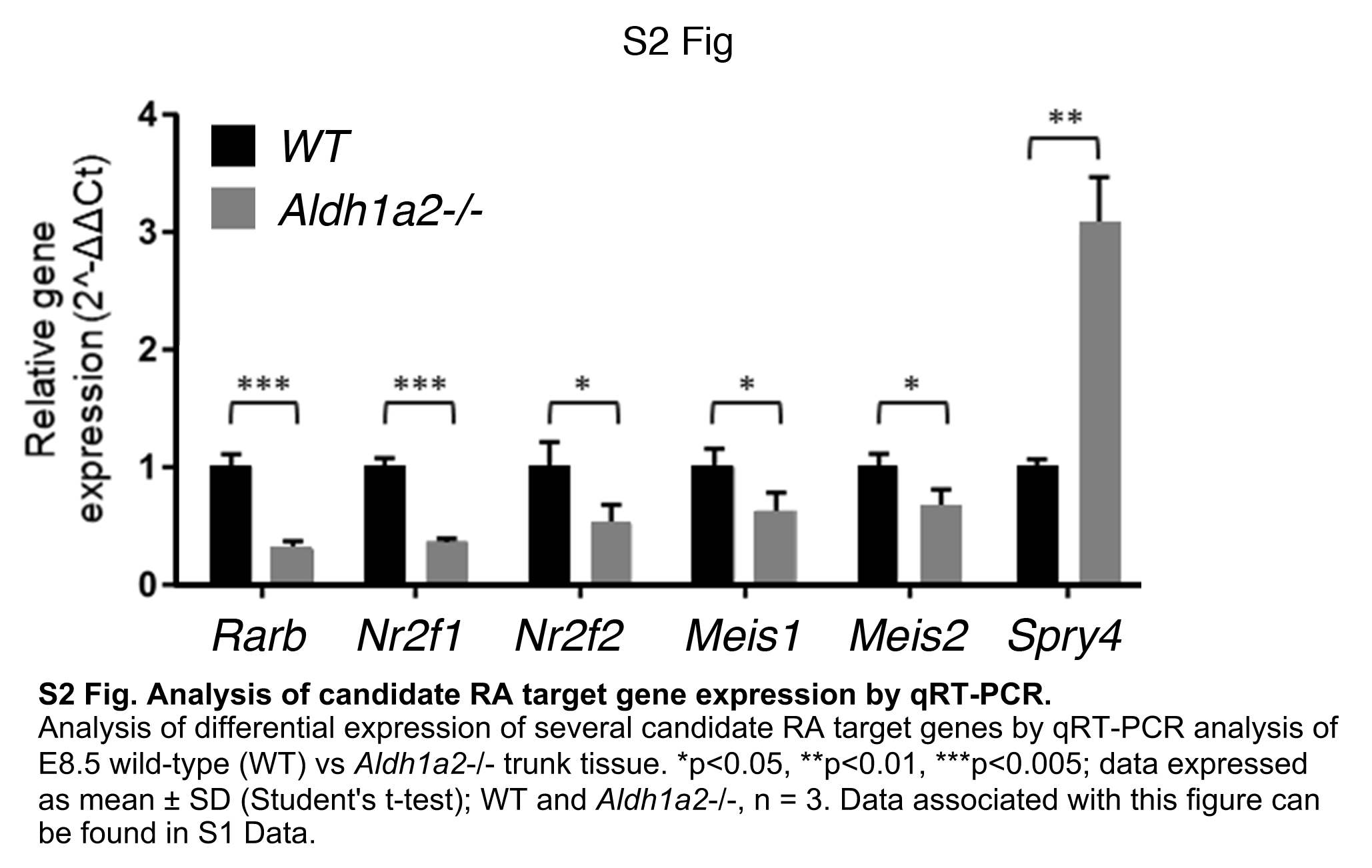

Supplement: S2 Fig — Analysis of differential expression of several candidate RA target genes by qRT-PCR analysis of E8.5 WT versus Aldh1a2-/- trunk tissue. *p < 0.05, **p < 0.01, ***p < 0.005; data are expressed as mean ± SD (Student t test); WT and Aldh1a2-/-, n = 3. Data associated with this figure can be found in S1 Data. Aldh1a2, aldehyde dehydrogenase 1A2; RA, retinoic acid; qRT-PCR, quantitative reverse transcription-polymerase chain reaction; SD, standard deviation; WT, wild type. (TIF) [file pbio.3000719.s006.tif]

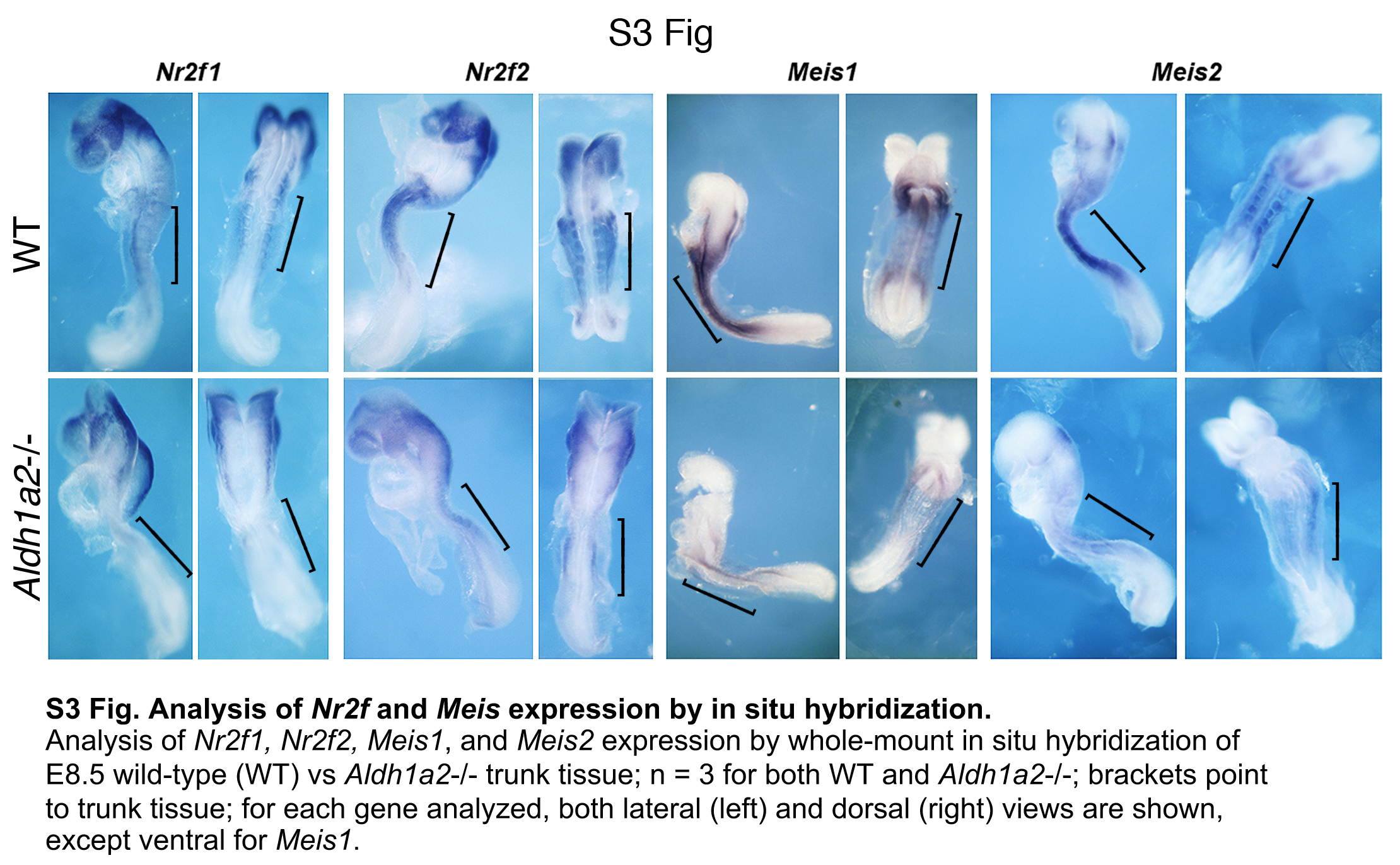

Supplement: S3 Fig — Analysis of Nr2f1, Nr2f2, Meis1, and Meis2 expression by whole-mount in situ hybridization of E8.5 WT versus Aldh1a2-/- trunk tissue; n = 3 for both WT and Aldh1a2-/-; brackets point to trunk tissue; for each gene analyzed, both lateral (left) and dorsal (right) views are shown, except ventral for Meis1. Aldh1a2, aldehyde dehydrogenase 1A2; E, embryonic day; Meis1, Meis homeobox 1; Meis2, Meis homeobox 2; Nr2f1, nuclear receptor 2f1; Nr2f2, nuclear receptor 2f2; WT, wild type. (TIF) [file pbio.3000719.s007.tif]
